# Whole genome sequencing and comparative genomics of closely related Fusarium Head Blight fungi: *Fusarium graminearum*, *F. meridionale* and *F. asiaticum*

**DOI:** 10.1186/s12864-016-3371-1

**Published:** 2016-12-09

**Authors:** Sean Walkowiak, Owen Rowland, Nicolas Rodrigue, Rajagopal Subramaniam

**Affiliations:** 1 Department of Biology, Carleton University, 1125 Colonel By Dr, Ottawa, Canada; 2 Agriculture and Agri-Food Canada, Government of Canada, 960 Carling Ave, Ottawa, Canada

**Keywords:** Fusarium Head Blight, Cereal pathogens, *Fusarium graminearum* species complex, Bioinformatics, Phylogenomics, Pan genome, Accessory genome

## Abstract

**Background:**

The *Fusarium graminearum* species complex is composed of many distinct fungal species that cause several diseases in economically important crops, including Fusarium Head Blight of wheat. Despite being closely related, these species and individuals within species have distinct phenotypic differences in toxin production and pathogenicity, with some isolates reported as non-pathogenic on certain hosts. In this report, we compare genomes and gene content of six new isolates from the species complex, including the first available genomes of *F. asiaticum* and *F. meridionale*, with four other genomes reported in previous studies.

**Results:**

A comparison of genome structure and gene content revealed a 93–99% overlap across all ten genomes. We identified more than 700 k base pairs (kb) of single nucleotide polymorphisms (SNPs), insertions, and deletions (indels) within common regions of the genome, which validated the species and genetic populations reported within species. We constructed a non-redundant pan gene list containing 15,297 genes from the ten genomes and among them 1827 genes or 12% were absent in at least one genome. These genes were co-localized in telomeric regions and select regions within chromosomes with a corresponding increase in SNPs and indels. Many are also predicted to encode for proteins involved in secondary metabolism and other functions associated with disease. Genes that were common between isolates contained high levels of nucleotide variation and may be pseudogenes, allelic, or under diversifying selection.

**Conclusions:**

The genomic resources we have contributed will be useful for the identification of genes that contribute to the phenotypic variation and niche specialization that have been reported among members of the *F. graminearum* species complex.

**Electronic supplementary material:**

The online version of this article (doi:10.1186/s12864-016-3371-1) contains supplementary material, which is available to authorized users.

## Background

Fungi belonging to the genus *Fusarium* are very diverse and occupy a wide variety of ecological niches. The genus is divided into several distinct species complexes, each with many species [1]. For example, the *Fusarium oxysporum* species complex is composed of a variety of species including plant and animal pathogens, decomposers and soil fungi [2–5]. The genomes of at least ten *F. oxysporum* isolates were sequenced in an attempt to delineate the genetic basis of niche specialization, and lineage specific regions within the genome were demonstrated to be important for pathogenicity in plants. For example, a supernumerary chromosome identified in *F. oxysporum* harbors virulence genes that confer pathogenicity to tomato [4].

In this study, we focused our attention on closely related individuals from the *Fusarium graminearum* species complex, which cause Fusarium Head Blight (FHB) disease of wheat. The species complex composed of several distinct species, including *F. graminearum*, *F. asiaticum*, and *F. meridionale* [6]. The genome of *F. graminearum* isolate PH-1 was the first genome available from the species complex, and has been re-examined as additional information on gene and genome structure has become available [7–10]. There have also been some large-scale genomics studies that have used this genome as a reference to study different biological phenomenon, such as genome wide association analyses of SNPs, as well as analyses of recombination events by restriction site associated DNA sequencing [11, 12]. Few studies have explored the genetic diversity of members of the *F. graminearum* species complex by performing whole genome assembly and comparative genetics; studies were largely focused on *F. graminearum sensu stricto* – a single lineage within the complex [7, 13, 14].

As part of the infection process, these fungi produce trichothecene mycotoxins that contribute to disease progression and accumulate in infected plant tissues [15]. The toxicity of *Fusarium* trichothecenes makes contaminated crops unsafe for consumption by animals [16]. The economic losses resulting from reductions in crop yield and grain quality has prompted significant amounts of research into understanding the genetic basis of toxin production by these fungi, which has been largely facilitated by the available genomic sequence of *F. graminearum* isolate PH-1 [7]. Phenotypic variations in toxin production, pathology on different hosts, development and morphology, and growth differences have been reported both among and between species of the *F. graminearum* species complex [17–19]. Currently, the only well-established virulence factor contributing to disease in wheat by *F. graminearum* and closely related species are the trichothecene mycotoxins [15, 20]. Allelic differences in genes as well as large-scale changes have been reported for the trichothecene gene cluster, with some genes occurring outside of the main cluster in separate regions of the genome [20]. For example, allelic differences in the biosynthetic gene *Tri8* are attributed to structural variation in the trichothecene deoxynivalenol (DON), namely 3-acetyldeoxynivalenol (3-ADON) and 15-acetyldeoxynivalenol (15-ADON) [21]. Similarly, allelic variation in *Tri1* is associated with the production of the alternate trichothecene NX-2, while *Tri13* and *Tri7* are associated with production of the trichothecene nivalenol [22, 23]. Although differences in genes that contribute to trichothecene variation are known, our current knowledge of other factors that lead to variations in disease profiles remains largely unknown. Information gathered from sequences of additional members of the *F. graminearum* species complex will lead to a better understanding of the genetic variations between isolates that are associated with reported phenotypic diversity.

This study reports on genomic and genetic differences among ten closely related members of the *F. graminearum* species complex that cause FHB. Six isolates were newly sequenced as part of this study (Accessions: NRRL 6101, NRRL 28720, NRRL 28721, NRRL 28723, NRRL 28336 and DAOM 180378) and four were sequenced previously (PH-1, DAOM 233423 or GZ 3639, DAOM 241165, and CS 3005) [7, 13, 14]. NRRL 6101, isolated from barley in Japan, was reported to be non-pathogenic in wheat and was categorized as *F. asiaticum* [17, 24]. NRRL 28720, NRRL 28721, and NRRL 28723 were isolated from maize in Nepal. While NRRL 28720 belongs to the species *F. asiaticum*, NRRL 28723 is of the related species *F. meridionale*, and the species classification for NRRL 28721 is still unclear and has been suggested to be a species hybrid (*F. asiaticum* x *F. meridionale*) [6, 17, 18, 25–27]. Further investigation with additional markers suggested that this isolate may be in a basal group within the *F. asiaticum* clade or belong within *F. meridionale* [6, 18]. The isolates PH-1, DAOM 233423, DAOM 241165, DAOM 180378, NRRL 28336, and CS 3005 belong to *F. graminearum sensu stricto*. With the exception of CS 3005, which was isolated in Australia, all other isolates were isolated in North America [6, 7, 13, 14]. PH-1 and DAOM 180378 were isolated from maize, NRRL 28336, DAOM 233423, and DAOM 241165 were isolated from wheat, and CS 3005 was isolated from barley. Both the trichothecene chemotype and pathology on wheat for each isolate has been reported (Table 1) [14, 17, 19, 21, 24, 28–30].

**Table 1:** Description of *Fusarium* Isolates Used in this Study

Accession	Species	Disease on Wheat^a, b^	Trichothecene Toxin^b^	Origin	
				Host	Location
PH-1	*F. graminearum*	High	15-ADON, DON	Maize	USA
CS 3005	*F. graminearum*	High	15-ADON, DON	Barley	Australia
DAOM 233423 (GZ 3639)	*F. graminearum*	High	15-ADON, DON	Wheat	USA
DAOM 241165	*F. graminearum*	High	3-ADON, DON	Wheat	Canada
DAOM 180378	*F. graminearum*	High	15-ADON, DON	Maize	Canada
NRRL 28336	*F. graminearum*	Medium	3-ADON, DON	Wheat	USA
NRRL 6101	*F. asiaticum*	None	3-ADON, DON	Barley	Japan
NRRL 28720	*F. asiaticum*	Medium	DON	Maize	Nepal
NRRL 28721	*F. meridionale*	Low	4A-NIV, NIV	Maize	Nepal
NRRL 28723	*F. meridionale*	Low	NIV	Maize	Nepal

^a^High = more than six florets infected, Medium = three to six florets infected, Low = infection on florets adjacent to inoculation site, None = no disease beyond the inoculation site

^b^Toxin analyses and pathology testing were performed in culture and/or on wheat [14, 17, 19, 21, 24, 28–30]

In this study, we compared the genome sequences of these ten closely related *Fusarium* isolates and identified genes that are part of the accessory genome and potentially involved in niche specialization within and between species.

## Methods

### *Fusarium* isolates, sequencing, and genome assembly

The isolates NRRL 28336, NRRL 28721, and NRRL 6101 were obtained from the collection at the Centraalbureau voor Schimmelcultures (CBS), in the Netherlands, while isolates NRRL 28720, NRRL 28723, and DAOM180378 were obtained from the collection at the Department of Agriculture Mycology (DAOM), in Canada. NRRL accession numbers are from the United States Department of Agriculture’s Agriculture Research Service Culture Collection. Total DNA was isolated from all the NRRL *Fusarium* isolates: 6101, 28336, 28720, 28721, 28723 and the DAOM isolate 180378 by the E.Z.N.A. Fungal DNA Mini Kit (Omega). DNA was sequenced according to manufacturer instructions using 74 bp reads from paired-end libraries by Illumina GAII sequencing. The sequencing reactions were performed by the Center for the Analysis of Genome Evolution and Function (CAGEF) at the University of Toronto, Canada. Sequence reads were imported into CLC Genomics Workbench. Prior to *de novo* assembly, reads were trimmed and short reads <20 bp were removed. Assemblies were optimized for contig length by adjusting the ‘word size’ parameter and keeping all other parameters at default settings. The optimal word sizes were determined to be 34, 41, 39, 38, 34, and 39 for NRRL 6101, NRRL 28720, NRRL 28723, NRRL 28336, and DAOM 180378, respectively. Average contig coverage was determined for each genome assembly and contigs that was 75% or lower than the average contig coverage and contigs shorter than 200 bp were removed from the assembly. The contigs were then reordered to the genome sequence of PH-1 from the MIPS database v3.2 by ABACAS [31]. The ordered contig sequences were uploaded to the GenBank database at NCBI (Table 2).

**Table 2:** Genome and Assembly Statistics of *Fusarium* Isolates Used in this Study

Isolate	Genome Size (Mb)	N50	Coverage (fold)	NCBI^a^ Accession	Genome Reference^b^
PH-1	36.6	5.37	10	AACM	Cuomo 2007 [7]
CS 3005	36.7	0.46	40	JATU	Gardiner 2014 [13]
DAOM 233423	36.5	0.17	86	LAJZ0	Walkowiak 2015 [14]
DAOM 241165	36.6	0.44	144	LAKA	Walkowiak 2015 [14]
DAOM 180378	36.4	0.16	53	LHUC	This study
NRRL 28336	36.7	0.40	36	LHUD	This study
NRRL 6101	36.5	1.21	111	LHTY	This study
NRRL 28720	36.4	0.35	36	LHTZ	This study
NRRL 28721	36.5	0.11	51	LHUA	This study
NRRL 28723	36.4	0.41	32	LHUB	This study

^a^National Center for Biotechnology Information

^b^Genomes were sequenced using Sanger, Illumina HiSeq 2000, and Illumina GAII sequencing for Cuomo 2007 [7], Gardiner 2014 [13], and Walkowiak 2015 [14], respectively

### Genomic alignments

The newly sequenced genomes, genome for PH-1 (MIPS v3.2), and sequences for CS 3005, DAOM 241165 and DAOM 233423 that were obtained from GenBank at NCBI were all used in separate pairwise genome alignments using the NUCmer and PROmer algorithms in the software package MUMmer [32, 33]. NUCmer is designed for aligning highly conserved regions of DNA, while PROmer aligns more divergent sequences. Since all matching and alignment routines are performed on the six frame amino acid translation of the DNA input sequence PROmer is more sensitive than NUCmer.

NUCMER was also used to extract the SNP and indel data, which was obtained by default parameters of the 1-to-1 algorithm in dnadiff [32, 33]. For each reference genome, the number of SNPs in each of the genomes was summed and averaged for each of the species to represent an average for each species. SNPs within conserved regions of the genome were obtained from PANSEQ with the following parameters: run mode = pan, fragment size = 500, percent identity cut-off = 90 and core genome threshold = 10 (i.e. sequences were present in all genomes) [34]. The resultant SNP alignment was imported into CLC Genomics Workbench for phylogenetic analyses using the maximum likelihood phylogeny tool, and the phylogeny constructed by the neighbor joining method and Jukes Cantor substitution model using 100 replicates; the bootstrap values at all nodes were 100%.

### Construction of pan and accessory genomes

Pan genome consists of full complement of genes from all ten genomes. The list was started by including all the genes from PH-1, then, genes from other genomes were compared to the current pan genome with BLASTn and added if the e-value was > 1E-10. The software program AUGUSTUS was used to predict genes from the six newly sequenced genomes [35]. The percentage alignment to PH-1 by MUMmer was used to select the order in which genomes were selected to contribute to the pan genome list going from greatest percent alignment to lowest. Genes in the pan genome that did not originate from PH-1 were annotated by Blast-2-Go using default parameters, which identified similar sequences in databases at NCBI, gene families/domains (InterPro), and gene ontology (GO) [36]. The accessory genome was created by comparing genes from the pan genome to each genome and included if they had a BLASTn e-value >1E-10 in at least one genome.

### Analysis of sequence variation within genes

For the analysis of sequence variation within genes, the number of positions that contained SNPs and indels from the dnadiff analysis in MUMmer were tabulated for the full length of the sequence for each gene. Orthologues of select PH-1 genes in the other genomes were obtained by BLASTn. The orthologues were aligned by MUSCLE using default parameters [37]. Genes containing a single orthologue in each genome were examined for nonsynonymous and synonymous substitution rates by Prank with the -codon -F options [38]. We then used CodeML to fit the M0, M7, and M8 models to study the overall nonsynonymous to synonymous substitution rate ratio (dN/dS) of these genes [39]. M0 is used to estimate global dN/dS ratios, while M7 and M8 are separate models that are compared and a likelihood ratio test showing the preference of M8 constitutes evidence of adaptive evolution [39].

## Results

### Genome assembly information and statistics

We performed whole genome sequencing of six fungal isolates (NRRL 6101, NRRL 28336, NRRL 28720, NRRL 28721, NRRL 28723 and DAOM 180378) from the *F. graminearum* species complex that are associated with FHB disease in plants (Table 1). The newly sequenced genomes, along with four previously sequenced isolates, were selected based on their incidence in various geographical locations, taxonomy/species, host isolation, toxin production and pathology [6, 17, 18, 26, 27] (Table 1). Overall, the ten sequenced genomes were comparable in size and structure when compared to the four previously characterized chromosomes of isolate PH-1 (Table 2) [7]. The sizes of the ten genomes ranged from approximately 36.4 to 36.7 Mb. The N50 values is defined as the contig length such that using equal or longer contigs produces half the bases of the genome, which ranged from 0.11 to 1.21 Mb for the six genomes sequenced by Illumina platforms; the genome coverages ranged from 32x to 144x (Table 2).

### Whole genome alignments of ten *Fusarium* pathogens within the *F. graminearum* complex

**Fig. 1:**
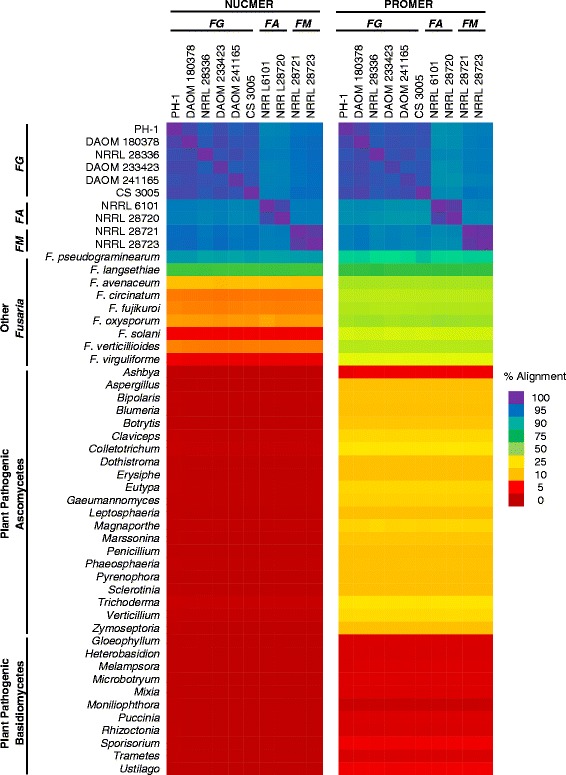
High concordance between genome alignments and taxonomy. Whole genomes were aligned between ten closely related isolates of *F. graminearum*, *F. asiaticum*, and *F. meridionale*, as well as 11 other *Fusaria*, individuals from 21 different *Ascomycete* genera, and individuals from 11 different *Basidiomycete* genera. Alignments were performed using two different algorithms, ‘NUCMER’ (*left*), which is designed for alignment of related sequences, and ‘PROMER’ (*right*), which is designed for alignment of distantly related sequences. Genomes are arranged by species; *F. graminearum* (*FG*), *F. asiaticum* (*FA*), and *F. meridionale* (*FM*). A non-linear full spectrum heat map is used to represent the percentage alignment to each query genome, where *red* has the lowest alignment and *violet* has the greatest alignment

To determine the degree of overlap among the ten genomes, we performed genome alignments of the ten genomes with one another (Table 2) and with 41 genomes of other plant pathogenic fungi using the NUCmer and PROmer algorithms in the software package MUMmer, (Fig. 1) [32]. A direct pairwise comparison with the sequence aligner NUCmer, which is designed for the alignment of closely related sequences, revealed at least 96.4% sequence alignment among the six *F. graminearum* genomes (PH-1, CS 3005, DAOM 233423, DAOM 241165, DAOM 180378, and NRRL 28336). Similarly, when the two *F. asiaticum* genomes (NRRL 6101 and NRRL 28720) were compared, 97.7% of the genomes aligned. Lastly, a comparison between the two *F. meridionale* genomes (NRRL 28721 and NRRL 28723) revealed a 99.0% alignment between them (Fig. 1). When we aligned the genomes of different species, *F. graminearum* and *F. asiaticum* had at least 93.1% sequence alignment, *F. graminearum* and *F. meridionale* had at least 94.4% sequence alignment, and *F. asiaticum* and *F. meridionale* had at least 93.8% sequence alignment. The ten genomes had between 90.9 and 92.0% alignment to *F. pseudograminearum* and 64.9–65.5% alignment to *F. langsethiae*, but only 3.5–12.8% aligned to other *Fusarium* genomes, and < 1% alignment to genomes from other plant pathogenic fungi (Fig. 1).

Analysis with PROmer, which is designed for the alignment of distantly related sequences, yielded results similar to those of NUCmer in pairwise comparisons of the ten genomes to one another and to *F. pseudograminearum* and *F. langsethiae* (Fig. 1). However, the alignment to other *Fusarium* species increased from 3.5–12.8 to 39.0–50.8%. Alignment to other phytopathogenic Ascomycetes increased from <1% to 3.4–26.1%, and alignment to phytopathogenic Basidiomycetes remained lower at 0.8–3.9% (Fig. 1). The concordance observed between genomes from our pairwise alignments agrees with taxonomic phylogenies suggested that *F. graminearum*, *F. asiaticum*, and *F. meridionale* are more closely related, while *F. pseudograminearum* and *F. langsethiae* are more distantly related, and that other Fusaria belong to even more distant taxonomic groups, followed by other genera and other phyla (Fig. 1). This agrees with the established evolutionary relationships suggested by other research studies [1, 6, 18, 40].

**Fig. 2:**
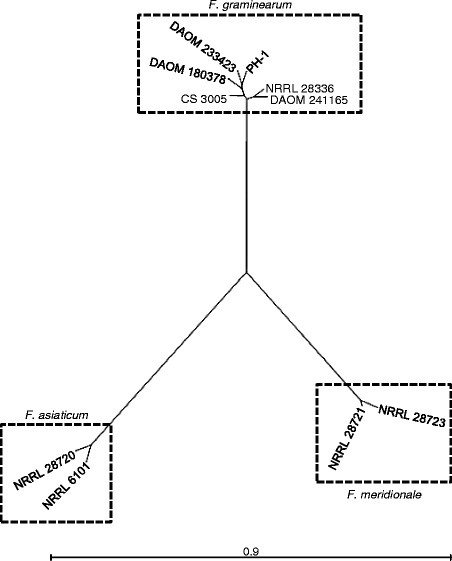
Phylogeny of FHB pathogens by whole genome alignment. A SNP and indel alignment of conserved genomic regions was performed by PANSEQ and was used to construct a maximum likelihood phylogeny by neighbor joining using 100 replicates. http://purl.org/phylo/treebase/phylows/study/TB2:S20221?x-accesscode=7489a1a68445a29618a659d472297db8&format=html

In addition to analyzing genetic overlap by direct pairwise alignments, we also identified SNPs and indels in all genomes, which were used to construct a maximum likelihood phylogenetic tree (Fig. 2). We used PANSEQ to identify a total of 704,566 SNPs and indels that differed among the ten genomes [34]. As observed, the ten isolates arranged according to their previously reported species assignments and were in agreement with the pairwise alignment data (Fig. 1). Together, the data indicate that PH-1, DAOM 233423, DAOM 180378, DAOM 241165, CS 3005, and NRRL 28336 are *F. graminearum sensu stricto*, while NRRL 6101 and NRRL 29720 are *F. asiaticum* [1, 6, 18, 40] (Fig. 2). The phylogeny also supports our pairwise genome alignment data which indicated that NRRL 28721 is more closely related to NRRL 28723 and may correspond to *F. meridionale* (Fig. 1). Interestingly, the two *F. graminearum* isolates that produce the trichothecene 3-ADON (DAOM 241165 and NRRL 28336) form a distinct genetic group from the four isolates that produce 15-ADON (PH-1, DAOM 233423, DAOM 180378, and CS 3005) (Fig. 2). This supports claims that individuals from these two chemotypes (3-ADON and 15-ADON) belong to distinctly separate genetic populations [41]. The *F. graminearum* isolate CS 3005, originating from Australia grouped in the same phylogenetic group as the other 15-ADON producers from North America, implying that it may belong to the same genetic population (Fig. 2).

### Regions of genomic variability in FHB fungi

**Fig. 3:**
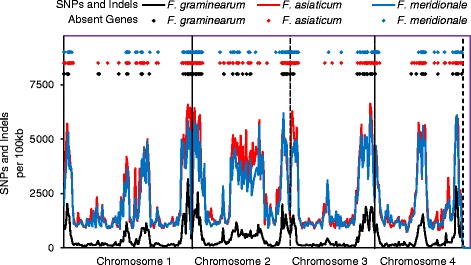
Genome sequence and gene content variability in PH-1 compared to other isolates. SNPs/indels and absent genes between genomes were determined separately, by whole genome alignments and BLASTn using PH-1 as a reference. The number of SNPs and indels per 100 kb were averaged for each species and are presented separately for *F. graminearum* (*black lines*), *F. asiaticum* (*red lines*), and *F. meridionale* (*blue lines*). Positions of absent genes whose sequences had a lowest expected value > 1E-10 by BLASTn for at least one member of the species are presented as sySNPsols; *F. graminearum* (*black diamonds*), *F. asiaticum* (*red diamonds*), and *F. meridionale* (*blue diamonds*). Chromosomes are demarked by vertical *dashed lines*

To visualize the genomic regions with sequence differences, the genomes were organized into 100 kb sections and the average number of SNPs and indels in the genomes of each species were plotted across the length of the four chromosomes using PH-1 as a reference (black line, Fig. 3). Generally, we observed that the average number of SNPs and indels were fewer within the species (black line) than genomes from *F. asiaticum* (red line) and *F. meridionale* (blue line) (Fig. 3). When a genome from a separate species was used as a reference, we observed that SNPs and indels were generally fewer in genomes within the same species than between genomes from different species (Additional file 1: Figure S1). Overall, we observed averages of 105–189 kb of SNPs and indels when comparing genomes from the same species and 796–893 kb of SNPs and indels when comparing genomes from different species. Sequence variation was co-localized at the ends of chromosomes and select regions within chromosomes, similar to what has been reported previously [14]. In addition, we also observed islands of increased nucleotide variation in specific genomes. For example, in the genomes of CS 3005, NRRL 28336, and NRRL 28720, we observed regions where the nucleotide variations increased compared to most other genomes (boxed, Additional file 1: Figure S1).

There is preponderance of evidence to suggest that niche adaptation, toxin production, and disease outcomes are attributed to differences in gene content and variation within genes [4, 14, 20–22, 42, 43]. We were interested to know if nucleotide variations were associated with genes absent among the ten genomes. AUGUSTUS was used to predict genes from the six genomes and gene sequences for isolates PH-1, DAOM 233423, DAOM 241165 and CS 3005 were obtained from previous studies [9, 13, 14, 35]. Gene conservation was determined by BLASTn using similar approaches to other studies [14]; genes were considered absent if they had the expected value > 1E-10 compared to other genomes. Our analysis showed that regardless of the reference genome, genes that are absent in *F. graminearum* (black symbols), *F. meridionale* (blue symbols), and *F. asiaticum* (red symbols) were all located in regions with high SNPs and indels (Fig. 3). We also identified genes that were unique to each isolate (purple symbols), which were also localized to regions of increased nucleotide variability (Additional file 1: Figure S1).

### Construction of the pan and accessory genomes of the *Fusarium* species complex

**Fig. 4:**
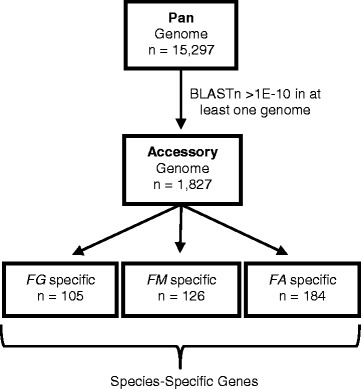
The accessory genome is a susbset of the pan-genome. The pan-genome, which incorporates the genes from all ten genomes, is composed of 15,297 genes, 1827 of which are accessory genes that are not conserved in all genomes. The accessory genome further divides into genes that are specific to each species, with 105 genes specific to *F. graminearum* (*FG*), 126 specific to *F. meridionale* (*FM*), and 184 specific to *F. asiaticum* (*FA*)

We constructed a pan genome by reiteratively appending genes of the PH-1 isolate from other genomes based on BLASTn expected values (e-value > 1E-10). This non-redundant gene list contained a total of 15,297 genes (Fig. 4). Of the 15,297 genes in the pan-genome 12% or 1827 genes had BLASTn hits with expected values > 1E-10 in at least one genome and was considered to be part of the accessory genome (Fig. 4, Additional file 2: Table S1). The accessory genome contributes to the genetic variability within and among species and might encode biochemical functions that are not essential for *Fusarium* growth, but confer selective advantages, such as adaptation to different niches or colonization of a new host [34].

The accessory genome was further parsed into genes that are unique to a species or to each isolate (Additional file 2: Table S2). Analyses identified a total of 105 species-specific genes that were present in all six *F. graminearum* isolates, but were absent in all genomes of the other two species (Fig. 4). Similarly, a total of 126 and 184 species-specific genes were found in *F. meridionale* and *F. asiaticum*, respectively (Fig. 4). Analysis of the accessory genome also revealed genes that are present in a single isolate, but absent in others (Uniquely Present, Table 3, Additional file 2: Table S2). We observed between 12 and 92 unique genes in each of the ten isolates (Uniquely Present, Table 3, Additional file 2: Table S2). In contrast, the *F. asiaticum* isolate NRRL 6101 had the most number of “uniquely absent” genes (34) and this isolate has been reported to be unable to infect wheat [17].

**Table 3:** Isolate-Specific Genes Identified from Accessory Genome

Isolate	Uniquely Present Genes^a^	Uniquely Absent Genes^b^
PH-1	14	13
DAOM 180378	18	8
NRRL 28336	92	2
DAOM 233423	18	10
DAOM 241165	12	1
CS 3005	14	6
NRRL 6101	65	34
NRRL 28720	72	29
NRRL 28721	18	2
NRRL 28723	22	7

^a^Uniquely present genes were considered present in the genome of the isolate and absent in genomes of all other isolates; absence was determined by having a BLASTn hit with the lowest expected value > 1E-10 in the genome of the isolate

^b^Uniquely absent genes were considered absent in the genomes of the isolate and present in the genomes of all other isolates; absence was determined by having a BLASTn hit with the lowest expected value > 1E-10 in the genome of the isolate

### The accessory genome reveals species and isolate-specific genes potentially involved in secondary metabolism and disease

Functional enrichment analyses of 105 *F. graminearum-*specific genes, 90 of which were originally identified in PH-1 by the MIPS FunCat database revealed 72 genes or 80% as unclassified. The remaining 18 genes were enriched in metabolic and defense pathways (Additional file 2: Table S3). The genes involved in defense included two aldehyde dehydrogenases, *FGSG_17538* and *FGSG_01759*, as well as *FGSG_17130* encoding a protein similar to pisatin demethylase cytochrome P450. Pisatin is a phytoalexin produced by plants as a defense molecule; detoxification of pisatin has been demonstrated by the action of demethylase genes from *F. solani* [44]. Analysis of 184 genes specific to *F. asiaticum* included secondary metabolic genes such as a non-ribosomal peptide synthetase (NRPS) APS1, which is involved in apicidin biosynthesis, and a polyketide synthase (PKS) PKS40 associated with W493 biosynthesis (Table 4) [42, 45, 46]. There were also 126 genes specific to *F. meridionale* and a gene (*g3644*) is annotated as a ‘pectin lyase fold virulence factor’ implicated in cell wall catabolism (Additional file 2: Table S4) [47]. Twelve heterokaryon or vegetative incompatibility genes were also identified as being species-specific; these gene classes are known to diversify in fungi and may have roles in speciation [48, 49].

**Table 4:** Secondary Metabolic Genes are Part of Accessory Genome

Gene Name	Source	Possible Function	Alternate Name(s)	Conservation^a^
Polyketide metabolism				
*FGSG_04694*	PH-1	polyketide synthase	PKS2	FG, FM
*FGSG_06540*	PH-1	polyketide cyclase		FG, FA
*g11843*	NRRL 28723	chalcone synthase b		FM, FA
*g6531*	NRRL 6101	polyketide enoyl_reductase		FA
*g1989*	NRRL 6101	polyketide synthase	PKS40	FA
*g21*	NRRL 28723	polyketide cyclase dehydrase protein		FM
*FG05_30424*	CS 3005	polyketide synthase	PKS52	FG, FA
*FG05_30565*	CS 3005	snoal-like polyketide cyclase family protein		FG, FM
*FG05_30492*	CS 3005	polyketide synthase		FG, FM, FA
*FG05_30491*	CS 3005	polyketide synthase		FG, FM, FA
*FG05_30490*	CS 3005	polyketide synthase		FG, FM, FA
*FG05_30489*	CS 3005	polyketide synthase	PKS43	FG, FM, FA
Non-ribosomal peptide metabolism				
*FGSG_10702*	PH-1	non-ribosomal peptide synthetase	NRPS17	FG
*g11745*	NRRL 6101	non-ribosomal peptide synthetase		FA
* g6644*	NRRL 6101	non-ribosomal peptide synthetase	APS1, NRPS31	FA
Terpene metabolism				
*FGSG_08181*	PH-1	terpene synthase		FG, FA
*g8968*	NRRL 28721	Tri5-like terpene synthase		FM, FA

^a^Gene was present in the genome of at least one isolate of *F. graminearum* (FG), *F. asiaticum* (FA), or *F. meridionale* (FM)

Our functional analysis was extended to isolate-specific genes. Of the 1827 genes from the accessory genome, 12–92 genes were present in a single isolate and were considered unique to that isolate (Table 3). The function of unique genes spanned a wide range of categories, including primary, secondary metabolism, as well as signal transduction. Examples include a glutamine synthase (g9634) from NRRL 28336 and a non-ribosomal peptide synthase (g11745) from NRRL 6101 (Table 4; Additional file 2: Tables S2 and S4). Among the 92 genes unique to NRRL 28336, nine are predicted to have kinase functions and could be involved in signal transduction (Additional file 2: Tables S2 and S4). A kinase unique to PH-1, *FGSG_11614*, has been disrupted and resulted in reduced DON production [50].

Genes that were uniquely absent in an isolate were also analysed (uniquely absent, Table 3). The isolate NRRL 6101 was reported to have limited virulence in wheat and contained the most number of uniquely absent genes (34, Table 3). These included two genes categorized by MIPS to be involved in cell rescue, defense and virulence, namely *FGSG_16080* predicted to encode a chitinase, and *FGSG_00132* with potential kinase function. The kinase *FGSG_00132* was previously target by reverse genetics, but no phenotype was observed in the mutant [50]. Another gene, *FGSG_08005* that was absent in the NRRL 28720 genome was predicted to be involved in isoprenoid metabolism, potentially contributing to secondary metabolic pathways (Additional file 2: Table S4).

Our analysis also showed that some of the genes in the accessory genome had irregular patterns of gene conservation across the ten genomes; many of these genes were associated with secondary metabolism (Table 4). Examples include *FGSG_04694*, a gene encoding polyketide synthase (PKS2), which was absent in both *F. asiaticum* genomes but was present in all *F. graminearum* and *F. meridionale* isolates (Table 4). Mutation of *PKS2* in *F. graminearum* showed reduced mycelial growth and a defect in virulence [51]. A polyketide cyclase, *FGSG_06540* showed poor conservation in both *F. meridionale* isolates, but was present in *F. graminearum* and *F. asiaticum* isolates [43]. Another example includes gene *g11843* from NRRL 28723 (*F. meridionale*), which shares similarity with *FAVG1_07768*, a gene from *F. avenaceum* that is present in the genomes of both *F. meridionale* isolates and the *F. asiaticum* isolate NRRL 6101, but is absent in all *F. graminearum* isolates. This gene was predicted to encode a chalcone synthase, which has been characterized in plants and is involved in the biosynthesis of defensive flavonoid compounds [52]. In addition to polyketide synthases, a metabolic gene *g8968* from NRRL 28721 (*F. meridionale*) shares identity with a terpene synthase gene, *Tri5*, which catalyzes the first step of trichothecene biosynthesis in *F. graminearum* and is essential for trichothecene biosynthesis and infection of wheat [15]. Interestingly, *g8968* did not have a positive BLASTn hit to the other *F. meridionale* genome NRRL 28723 but had positive BLASTn hits to the *F. asiaticum* genomes NRRL 6101 and NRRL 28720. Upon closer examination, g8968 showed only 21.8% protein identity to Tri5 from the *F. graminearum* isolate PH-1, but 95% protein identity to FAVG1_13210 from *F. avenaceum* [53]. A true homologue of Tri5 (g5480) with >97% protein identity was identified in the *F. meridionale* isolate NRRL 28721. Despite the low identity to Tri5, g8968 was predicted to contain a Tri5 domain by Interpro and Pfam (e-value 5.3E-15). It is unclear if the protein from this gene acts on substrates from the trichothecene biosynthetic pathway and contributes to their production, or if the protein is involved in the production of other molecules.

In addition to individual genes involved in secondary metabolism, accessory genes with irregular patterns of conservation also extended to gene clusters. For example, a gene cluster composed of five genes was absent in the *F. meridionale* genomes, but is present in *F. graminearum* and *F. asiaticum* isolates (Table 4) [42, 43]. The genes within this cluster include *FGSG_08181*, which is predicted to encode a terpene synthase, *FGSG_08182*, which encodes a putative transcription factor and three putative cytochrome P450 genes *FGSG_17088*, *FGSG_08183*, and *FGSG_08187* [42, 43]. Genes from this cluster have been reported to be expressed during infection in different cereal hosts [54]. Similarly, another gene cluster (*FG05_30489 - FG05_30492*) from the *F. graminearum* isolate CS 3005 from Australia was absent in the 15-ADON producing isolates of *F. graminearum* from North America, but was present in all other genomes (Table 4). The gene *FG05_30489* with similarity to a polyketide synthase gene *PKS43* was also present in the *F. avenaceum* and *F. equiseti* genomes [42].

In addition to the genes potentially involved in secondary metabolism, there were other genes from the accessory genome with irregular conservation patterns that have been shown to contribute to fecundity and disease by the fungi (Table 4). A study that targeted transcription factors determined that accessory genes *FGSG_08182* and *FGSG_11561* contributed to disease and spore production, respectively [55]. A separate study that focused on kinases determined that the accessory genes *FGSG_07812*, *FGSG_11614*, and *FGSG_02153* all contributed to toxin production while *FGSG_03146* contributed to spore production and disease [50]. Disruption of a possible phosphatase regulatory subunit *FGSG_11641* has also been shown to reduce disease accumulation [56]. Together, the accessory genome appears to contain genes that contribute to metabolic processes, as well as other processes potentially involved in development or disease.

### Highly polymorphic genes in common genome include alleles, pseudogenes and genes under diversifying selection

**Fig. 5:**
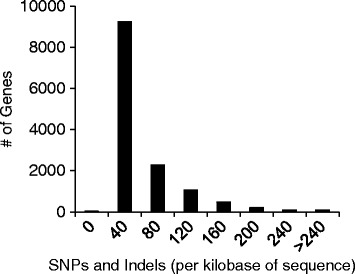
Sequence variability within common genes. Only 163 genes contain greater than 20% sequence variation. Genetic variability of genes was assessed based on SNPs and indels from the genome alignments

Genes not considered part of the accessory genome was identified to contain high levels of sequence variation. We identified a total of 163 genes that were highly variable with >200 SNPs or indel sites per kb (Fig. 5, Additional file 2: Table S5). Since 110 of the163 genes originated from PH-1, the MIPS FunCat database (http://mips.helmholtz-muenchen.de/funcatDB/) was used to detect functional enrichment of gene categories (Additional file 2: Table S6). The analysis revealed that the majority of the genes (73.6%) are functionally unclassified, compared to 52.3% for all genes from PH-1. Functional categories that were determined to be enriched compared to the rest of the genome (*p*-value < 0.05) included motility, energy, metabolism, and cell migration (Additional file 2: Table S6). Select genes that exhibited high frequency of SNPs and indels were inspected in greater detail, these included *Tri8*, three putative transcription factors, two proteins with nodulin-like domains and three putative heterokaryon incompatibility genes.

Allelic variation in Tri8 is attributed to the production of either 15-ADON or 3-ADON [21]. Multiple sequence alignment by MUSCLE indicated that within a specific chemotype, such as 3-ADON producing isolates, *Tri8* had greater than 95% sequence identity in the coding regions. Comparison of the coding sequences between chemotypes showed only 84–89% sequence identity. Although, no acetylated form of DON has been shown to be produced by NRRL 28720 isolate, *Tri8* from this isolate showed 97% identity to other15-ADON producing isolates [17]. Thus, high alignment of *Tri8* suggests that the high variation in this gene may be associated with allelic variation within chemotypes.

Three genes *FGSG_13457*, *FGSG_08954*, and *FGSG_10508* that encode transcription factors have been previously targeted for functional analysis [55]. Disruption of *FGSG_13457* resulted in no apparent phenotypic difference in toxin production or disease, while no mutants were obtained for *FGSG_08954* and *FGSG_10508* [55]. Closer inspection of *FGSG_08954* in the ten genomes revealed that the gene may have a small deletion and a premature stop codon in NRRL 28720 and duplicate copies in DAOM 241165. The two other transcription factors FGSG_13457 and FGSG_10508 were subjected to diversifying selection analysis by CodeML to assess overall nonsynonymous to synonymous substitution (*dN/dS*) rate of these genes. With the M0 model, the *dN/dS* ratios for *FGSG_13457* and *FGSG_10508* were estimated to be 0.221 and 0.408, respectively, and application of the two site model (M7 and M8) did not provide evidence of diversifying selection (Additional file 2: Table S7).

Two genes encoding putative nodulin-like domains, *FGSG_03381* and *FGSG_03550*, were also found to have high sequence variation; nodulin-like domains are often found in plant proteins and are implicated in plant-microbe interaction [57]. The orthologue of *FGSG_03550* spanned a region with ambiguous nucleotides for four of the genomes or was at the very end of a contig, the gene also contained a 24 bp insertion in the two *F. asiaticum* genomes. The *dN/dS* ratio of *FGSG_03381* was 0.355, and the two site model indicated no evidence for adaptive evolution (Additional file 2: Table S7).

Lastly, three putative heterokaryon incompatibility genes *FGSG_10601*, *FGSG_08120* and *FGSG_08144* with potential roles in non-self-recognition were identified to have high sequence variation [48, 49]. *FGSG_08120* was determined to have a premature stop in NRRL 6101, while the closest orthologue of *FGSG_08144* in NRRL 6101 had an identity <50 to the orthologues. Analysis of diversifying selection for *FGSG_10601* showed a dN/dS ratio of 0.708 and was the only gene from the four investigated that showed significant evidence of adaptive evolution (with a *p*-value *<* 0.01) by the two site model (Additional file 2: Table S7).

Altogether, the increased sequence variation in select common genes may be associated with allelic variation (*Tri8*) or mutations resulting in premature stop codons resulting in a pseudogene (*FGSG_08120* and *FGSG_08954*). In addition, *FGSG_10601* was determined to be under adaptive evolutionary pressures that may have contributed to diversification of this gene.

## Discussion

Fusarium Head Blight fungi are globally distributed and isolates are known to exhibit phenotypic diversity in their morphology, growth and development, production of toxins and other secondary metabolites, and pathogenicity towards specific hosts [17]. DNA-based phylogenetic analyses using gene markers have allowed us to identify distinct species and genetic populations of these fungi. However, some isolates have proven difficult to classify using marker analyses and the biological mechanisms underlying phenotypic diversity remain unsettled [6, 17, 18, 25–27]. Genome sequencing of additional isolates provided the opportunity to better understand and confirm the genetic relationship between fungal isolates, and identified gene candidates that could contribute to phenotypic differences and niche adaptation. Similar approaches were successfully applied to the *F. oxysporum* species complex; for example, genomic comparison of *F. oxysporum* isolates identified gene expansion that may be associated with pathogenicity towards banana, as well as genes associated with infection in melon [2, 5].

A comparative genomics approach used in this study was effective at better resolving the genetic relationship among fungal species and isolates. In pairwise alignments, the genomes of the ten closely related Fusaria exhibited high levels of identity, particularly within species. The genomes from isolates of the *F. graminearum* species complex also had greater alignment to *F. pseudograminearum* and *F. langsethiae*, than to genomes from other Fusaria, or other genera altogether. These findings confirm the previously established phylogenetic relationships among these closely related *Fusarium* to each other and other fungi [4, 17, 18]. Interestingly, on average, *F. meridionale* shared more genes and showed high levels of identity than *F. asiaticum* to *F. graminearum*. Based on these findings, *F. meridionale* and *F. graminearum* may be more closely related than *F. asiaticum* and *F. graminearum*. In addition to providing insights into the relationships between species, we were also able to better resolve species placement of NRRL 28721, which was difficult to determine using marker analyses. Marker analyses had suggested that NRRL 28721 is either *F. asiaticum* or *F. meridionale* or a species hybrid [6, 17, 18, 25–27]. Analyses of NRRL 28723 and NRRL 28721genomes showed high level of identity (99.0%). In combination of with SNPs and indels analyses, we suggest that NRRL 28721 and NRRL 28723 isolates are closely related and may be of the same species, *F. meridionale* (Fig. 2). Similar analyses also enabled us to separate 3-ADON or 15-ADON genetic populations within *F. graminearum* species complex that have been reported by others [41].

This study provided insights into the relationships between isolates with known differences in geographical distribution, species and population backgrounds. For example, CS 3005, an isolate from Australia grouped within the same population as the 15-ADON producers in North America. This suggested that this Australian isolate may be more related to the 15-ADON producers rather than the 3-ADON producers from North America. Furthermore, the study suggests that genetic populations are globally distributed, and that geographical barriers may not play a significant role in the spread of these fungi. The presence of different species such as *F. asiaticum* and *F. meridionale* in Nepal, or genetic populations of the same species, such as 3-ADON and 15-ADON populations of *F. graminearum* in North America that cause similar diseases indicates that different populations and species can coexist and may have distinguishable traits that allow them to occupy slightly different niches than other populations or species. For example, environmental and host conditions have been demonstrated to affect disease outcomes by 3-ADON and 15-ADON producers of *F. graminearum* [58]. Further monitoring will better resolve the geographical distribution of these populations/species and the stability of the population and community structure over time. This may be particularly important when we consider the environmental consequences of climate change.

In addition to providing insights into the genetic relationship between isolates, our comparative genomics analyses also provided perspective into the conservation and evolution of genes that may contribute to phenotypic differences among these pathogens. We observed a high degree of conservation (95 to 99%) of genes among the ten genomes; highest within species than between species (Additional file 3: Figure S2). Analyses of the pan genome indicated that the two 3-ADON producing strains of *F. graminearum* (NRRL 28336 and DAOM 241165) had the fewest number of absent genes (914 and 990, respectively). In contrast, the two *F. asiaticum* isolates NRRLs 6101 and 28720 had the greatest number of absent genes, 1126 and 1146, respectively, when compared to the pan genome (Additional file 2: Table S2). Further study across additional isolates would be required to determine if there is gene expansion or loss in populations or species of these fungi.

Many of the genes from the accessory genome were predicted to be involved in metabolism. This is particularly important because *Fusarium* species are well known for their ability to make a wide array of secondary metabolites, including DON, which has been studied for its role as a virulence factor during plant infection and for its toxic effects in humans [15]. Based on the sequence, *F. graminearum* isolate PH-1 is reported to have at least 16 known PKS genes and 19 known NRPS and other metabolic genes [42, 43]. We discovered that some of these genes, such as *PKS2*, *PKS52* and *NRPS17* that have described previously in *F. graminearum* are absent in some of the genomes [42]. We also identified genes in some of the ten genomes, such as *NRPS31*, that were previously not reported in the *F. graminearum* species complex (Table 4) [42]. These analyses underscore the potential importance of secondary metabolites in host and niche adaptation and future studies should focus on the discovery of metabolites produced by these enzymes.

**Fig. 6:**
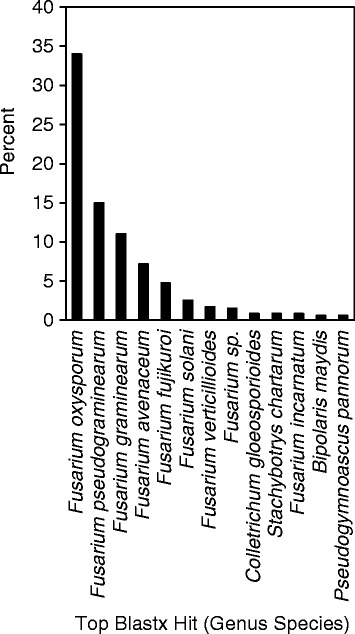
Top BLASTx hit of genes from the pan-genome not from PH-1 or CS 3005. The top 13 genus/species that match to the genes are presented in descending order and are presented as a percentage of genes; all other species/genus had less than five genes (0.5% of genes) that were a top hit to that genus/species

Genes to be species or isolate specific were annotated by Blast-2-Go based on sequence similarity to genes from other Fusaria (Fig. 6). A total of 1061 gene sequences were annotated from the new genomes (excluding CS 3005 and PH-1) and 361 or 34% of these had a top tBLASTx hit in *F. oxysporum*, while the next top hits were in *F. pseudograminearum* (15%), *F. graminearum* (11%), *F. avenaceum* (7%), *F. fujikuroi* (5%), *F. solani* (3%), and *F. verticillioides* (2%) (Fig. 6). All other genus/species that had more than 0.5% of genes BLASTx hits were from other Fusaria or other Ascomycete genera, including *Colletotrichum*, *Stachybotrys*, *Bipolaris*, and *Pseudogymnoascus*. This indicates that many genes from the accessory genome are conserved across the genus, but may be lost or acquired, depending on selective pressures from the environment or plant host. This has been previously described as an attribute of pathogen effectors that are involved in the evolutionary interactions between pathogen and host.

Such conservation of accessory genes within the genus also suggest that horizontal gene transfer may play a significant role in the biology of *Fusarium*. This is supported by our observations of genes that were absent across isolates and were co-localized in regions of the genome with increased nucleotide variability (Fig. 3). The co-localization of divergent genetic regions and genes that are absent among related genomes is consistent with what has been reported previously for *Fusarium* species [4, 14, 59]. Co-localization may suggest that genes were acquired or lost as clusters; multiple gains or losses of gene clusters have been suggested for the secondary metabolite gene cluster responsible for fumonisin biosynthesis in *Fusarium* [60]. Lineage specific chromosomes and large scale horizontal gene transfer events, such as the transfer of an entire chromosome that have been reported in *F. oxysporum*, are yet to be reported for members of the *F. graminearum* species complex [4]. However, small scale horizontal transfer events have been suggested between *F. graminearum* and other fungi [43]. Horizontal gene transfer has also been suggested between bacteria and *F. pseudograminearum*, which is also within the *graminearum* species complex and is reported to cause disease on the roots of cereals [59]. Closer inspection of the presence, location, and orientation within the genomes of other organisms could shed light on their history and origin.

In addition to accessory genes contributing to genetic differences amongst the genomes, we also identified common genes that are variable and could contribute to niche adaptation and disease. Common genes involved in plant-pathogen interaction that are recognized by the host are selected against but in some cases cannot be shed; instead, these genes modify their sequence in order to avoid recognition by the host [61]. Our analyses identified 163 common genes with more than 20% sequence variability present among genomes. This included *Tri8*, which has reported allelic variation and contributes to the structural variation that exists between 3-ADON and 15-ADON toxins [21]. Other genes, such as *FGSG_08120* and *FGSG_08954* may have acquired mutations that resulted in pre mature stop codons, resulting in pseudogenes. In addition, analysis of synonymous and nonsynonymous mutation rates of *FGSG_10601* indicated that this gene may be under diversifying selection. A comparative analysis of three *Fusarium* species identified genes from *F. graminearum* that may be under diversifying selection [62]. These included *FGSG_03550*, *FGSG_03859*, *FGSG_05819*, *FGSG_10508*, *FGSG_11067*, *FGSG_12487*, *FGSG_13517*, *FGSG_04520*, *FGSG_10636*, and *FGSG_15331*, which were also identified to have >20% sequence variation between genomes in our study [62]. In contrast, our analyses of *FGSG_10508* among the *F. graminearum* species complex did not find significant evidence for diversifying selection, possibly due to the shorter evolutionary distance between the genomes we investigated. Nevertheless, further investigation of these highly variable common genes as well as the accessory genes could implicate these genes with phenotypic differences or known adaptive processes.

## Conclusions

Comparative genomics has enabled us to place ten isolates from the *F. graminearum* species complex into their respective species or genetic populations. A construction of a pan and accessory genome has also given us insights into the genetic diversity among members of the *F. graminearum* species complex. Further investigation of the roles of both accessory genes and highly variable common genes with respect to niche adaptation is required. As more genomes of this species complex become available, a larger comparative study will allow for further characterization of the genetic diversity amongst these economically important pathogenic fungi.

## Additional files

Additional file 1: Sequence and gene content variability between genomes. Description: SNPs/indels and absent genes between genomes were determined separately, by whole genome alignments and BLASTn, using each genome as a reference. a, *F. graminearum* genomes were used as a reference. b, *F. meridionale* genomes are used as a reference. c, *F. asiaticum* genomes are used as a reference. The number of SNPs and indels per 100 kb between the reference genome and the average for each species is presented separately for *F. graminearum* (black lines), *F. asiaticum* (red lines), and *F. meridionale* (blue lines). The minimum and maximum for each plot is 0 and 10,000 nucleotide positions. Positions of absent genes whose sequences had a lowest expected value > 1E-10 by BLASTn for at least one member of the other species are presented as symbols; *F. graminearum* (black diamonds), *F. asiaticum* (red diamonds), and *F. meridionale* (blue diamonds). Absent genes in all other members of all species were considered unique genes (purple squares). Some regions of SNPs /indels that are greater in a specific reference genome than in other reference genomes are highlighted (dotted box). (DOCX 635 kb)
Additional file 2: Summary tables for gene conservation, variation, annotation, and functional enrichment analyses. Description: **Table S1.** the accessory genome is a subset of the pan genome. **Table S2.** components of the accessory genome that are species and isolate specific. **Table S3.** functional categories of *F. graminearum* species-specific accessory genes. **Table S4.** genes from the pan genome not from PH-1 were annotated by Blast-2-Go to be involved in a wide array of processes. **Table S5.** common genes with >20% sequence variation. **Table S6.** common genes that are variable are mostly of unknown function. **Table S7.** parameter values and log likelihood scores of codon substitution models. (XLSX 2162 kb)
Additional file 3: Percent gene overlap between genomes. Description: The percentage of genes from the query genome in the reference genome are presented in a greyscale heatmap, where white represents 95% conservation and black represents 100% conservation. Genes were considered absent if they had a lowest BLASTn e-value > 1E-10. Genomes are arranged by species; *F. graminearum* (*FG*), *F. asiaticum* (*FA*), and *F. meridionale* (*FM*). (DOCX 28 kb)

## Abbreviations

15-ADON: 15-acetyldeoxynivalenol
3-ADON: 3-acetyldeoxynivalenol
CAGEF: Center for the Analysis of Genome Evolution and Function
CBS: Centraalbureau voor Schimmelcultures
DAOM: Department of Agriculture Mycology
DON: Deoxynivalenol
FA: *Fusarium asiaticum*
FG: *Fusarium graminearum*
FHB: Fusarium Head Blight
FM: *Fusarium meridionale*
GO: Gene ontology
Indel: Insertion and/or deletion
kb: Kilo base pairs
Mb: Mega base pairs
NRPS: Non-ribosomal peptide synthase
ORDC: Ottawa Research and Development Centre
PKS: Polyketide synthase
SNP: Single nucleotide polymorphism
