# Supplementary material for: Whole genome sequencing and comparative genomics of closely related Fusarium Head Blight fungi: Fusarium graminearum, F. meridionale and F. asiaticum
Source: BMC Genomics. 2016 Dec 9;17:1014. doi: 10.1186/s12864-016-3371-1 (PMC5148886; doi:10.1186/s12864-016-3371-1)
Supplement: Additional file 1: — Sequence and gene content variability between genomes. Description: SNPs/indels and absent genes between genomes were determined separately, by whole genome alignments and BLASTn, using each genome as a reference. a, F. graminearum genomes were used as a reference. b, F. meridionale genomes are used as a reference. c, F. asiaticum genomes are used as a reference. The number of SNPs and indels per 100 kb between the reference genome and the average for each species is presented separately for F. graminearum (black lines), F. asiaticum (red lines), and F. meridionale (blue lines). The minimum and maximum for each plot is 0 and 10,000 nucleotide positions. Positions of absent genes whose sequences had a lowest expected value > 1E-10 by BLASTn for at least one member of the other species are presented as symbols; F. graminearum (black diamonds), F. asiaticum (red diamonds), and F. meridionale (blue diamonds). Absent genes in all other members of all species were considered unique genes (purple squares). Some regions of SNPs /indels that are greater in a specific reference genome than in other reference genomes are highlighted (dotted box). (DOCX 635 kb) [file 12864_2016_3371_MOESM1_ESM.docx]

SNPs and Indels
per 100kb

0

10000

0

10000

0

10000

0

10000

10000

0

SNPs and Indels
per 100kb

PH-1

DAOM 233423

CS 3005

DAOM 241165

**a**

**b**

NRRL 28336

DAOM180378

10000

0

10000

SNPs and Indels:

Absent Genes:

SNPs and Indels
per 100kb

0

10000

NRRL 28721

**c**

NRRL 28723

10000

0

10000

0

NRRL 6101

NRRL 28720

0

Chromosome 1 Chromosome 2 Chromosome 3 Chromosome 4
