# Supplementary material for: Whole genome sequencing and comparative genomics of closely related Fusarium Head Blight fungi: Fusarium graminearum, F. meridionale and F. asiaticum
Source: BMC Genomics. 2016 Dec 9;17:1014. doi: 10.1186/s12864-016-3371-1 (PMC5148886; doi:10.1186/s12864-016-3371-1)
Supplement: Additional file 3: — Percent gene overlap between genomes. Description: The percentage of genes from the query genome in the reference genome are presented in a greyscale heatmap, where white represents 95% conservation and black represents 100% conservation. Genes were considered absent if they had a lowest BLASTn e-value > 1E-10. Genomes are arranged by species; F. graminearum (FG), F. asiaticum (FA), and F. meridionale (FM). (DOCX 28 kb) [file 12864_2016_3371_MOESM3_ESM.docx]

|  | PH-1 | | DAOM 180378 | | NRRL 28336 | | DAOM 233423 | | DAOM2 41165 | | CS 3005 | | NRR L6101 | | NRRL 28720 | | NRRL 28721 | | NRRL 28723 | |  |
| --- | --- | --- | --- | --- | --- | --- | --- | --- | --- | --- | --- | --- | --- | --- | --- | --- | --- | --- | --- | --- | --- |
| PH-1 | | 100.0 | | 99.3 | | 98.8 | | 99.3 | | 99.0 | | 99.2 | | 96.2 | | 96.2 | | 97.3 | | 97.4 | |
| DAOM 180378 | | 99.3 | | 100.0 | | 98.9 | | 99.2 | | 99.1 | | 99.0 | | 96.1 | | 96.0 | | 97.3 | | 97.5 | |
| NRRL 28336 | | 97.8 | | 97.9 | | 100.0 | | 97.7 | | 98.6 | | 98.0 | | 95.6 | | 95.5 | | 96.4 | | 96.5 | |
| DAOM 233423 | | 99.4 | | 99.4 | | 98.9 | | 100.0 | | 99.1 | | 99.2 | | 96.3 | | 96.2 | | 97.5 | | 97.6 | |
| DAOM 241165 | | 98.6 | | 98.8 | | 99.3 | | 98.6 | | 100.0 | | 98.8 | | 96.1 | | 96.0 | | 97.1 | | 97.2 | |
| CS 3005 | | 99.0 | | 98.9 | | 98.9 | | 98.9 | | 99.0 | | 100.0 | | 96.2 | | 96.1 | | 97.3 | | 97.3 | |
| NRRL 6101 | | 96.7 | | 96.7 | | 97.2 | | 96.7 | | 97.0 | | 96.9 | | 100.0 | | 98.6 | | 96.7 | | 96.7 | |
| NRRL 28720 | | 96.8 | | 96.7 | | 97.1 | | 96.7 | | 97.1 | | 96.9 | | 98.7 | | 100.0 | | 97.0 | | 96.8 | |
| NRRL 28721 | | 97.5 | | 97.6 | | 97.7 | | 97.6 | | 97.8 | | 97.8 | | 96.4 | | 96.6 | | 100.0 | | 99.5 | |
| NRRL 28723 | | 97.6 | | 97.7 | | 97.8 | | 97.7 | | 97.8 | | 97.7 | | 96.4 | | 96.3 | | 99.5 | | 100.0 | |

Reference Genome

*FG FA FM*

|  |  |
| --- | --- |
| % Gene Conservation | |
|  | |
|  | 100 |
|  | 99 |
|  | 98 |
|  | 97 |
|  | 96 |
|  | 95 |
|  |  |

*FM FA FG*

Query Genome Genes
